# Impact of gate electrode on free chlorine sensing performance in solution-gated graphene field-effect transistors†

**DOI:** 10.1039/d3ra07692j

**Published:** 2024-03-06

**Authors:** Masato Sugawara, Takeshi Watanabe, Yasuaki Einaga, Shinji Koh

**Affiliations:** a Department of Electrical Engineering and Electronics, Aoyama Gakuin University 5-10-1 Fuchinobe, Chuo-ku Sagamihara 252-5258 Japan twatanabe@ee.aoyama.ac.jp; b Department of Chemistry, Keio University 3-14-1 Hiyoshi, Kohoku-ku Yokohama 223-8522 Japan

## Abstract

Free chlorine is widely used to disinfect water used for drinking and food processing. This requires highly sensitive, simple, and capable continuous-measurement sensors to enable the concentration of free chlorine in water to be monitored and controlled. Free chlorine sensors based on solution-gated graphene field-effect transistors (GFETs) are a suitable platform for highly sensitive and continuous measurements. However, their sensing mechanisms require further elucidation to improve their performance. In this study, we focused on the gate electrode and investigated its influence on the sensing performance. Using the free chlorine sensor based on the solution-gate GFET, we showed that the Dirac point voltage in the transfer curve changed significantly as the free chlorine concentration changed, and the electric double-layer capacitance of the gate electrode decreased. Furthermore, we demonstrated that a solution-gated GFET using graphene or boron-doped diamond as the gate electrode could be used to detect changes in the free chlorine concentration in the concentration range of tap water. The sensing performance in the low concentration range benefits from the wide potential window of carbon-based electrodes, which do not have electrochemically active sites. Using these carbon-based materials as gate electrodes, GFETs have the potential to be used as durable sensors that are resistant to surface fouling and oxidation.

## Abstract 2

We investigated the role of gate electrodes in solution-gated graphene field-effect transistors for sensing free chlorine. Graphene and boron-doped diamond exhibit suitable electrochemical properties for gate electrodes.

## Graphical abstract

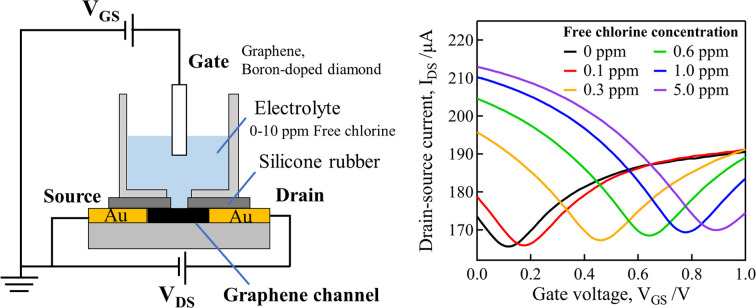

## 1 Introduction

Free chlorine, comprising Cl_2_(g), HClO(aq), and ClO^−^(aq), is widely utilized as a disinfectant and deodorizer in various aspects of our daily lives. It plays a crucial role in eliminating pathogens in drinking water, swimming pool water, and industrial wastewater treatment processes.^1,2^ The presence of residual chlorine in drinking water serves as a vital safety indicator. In regions where access to safe drinking water is a pressing challenge, the importance of maintaining sufficient levels of free chlorine is heightened. The World Health Organization (WHO) recommends a minimum residual chlorine concentration of 0.2–0.5 mg Cl per L (ppm) at the point of delivery.^3^ This guideline not only underscores the critical function of free chlorine in ensuring effective disinfection but also highlights its pivotal role in providing microbiologically safe drinking water, especially in resource-limited areas. The measurement of free chlorine is emerging as an indispensable practice for safeguarding public health, underscoring its significance within the broader global efforts to enhance access to safe drinking water.^4^ An overly low free residual chlorine concentration in the water would not destroy pathogenic bacteria and viruses, and disinfection would be inadequate.^5^ Conversely, excess free residual chlorine in the water would react with many organic substances present in the water to generate byproducts such as trihalomethane, which may adversely affect human health and the environment.^6,7^ The WHO has established a health-based guideline value of 5 ppm in drinking water.^3^ It is therefore crucial to carefully monitor the concentration of free residual chlorine in treated water from various sources, including drinking water, pool water, and reusable wastewater. A highly sensitive, accurate, and continuous analytical method is therefore necessary to ensure that the concentration is neither too low nor too high.

To date, various analytical methods to measure free chlorine, including spectrophotometry,^8^ liquid chromatography,^9^ and ion chromatography,^10^ have been reported. However, each method has its particular drawbacks, amongst which are low sensitivity, low selectivity, and the need for bulky and expensive analytical equipment. As a result, two measurement methods that are simpler than these methods have been employed in practice: the colorimetric or photometric methods, which uses *N*,*N*′-diethyl-*p*-phenylenediamine (DPD) and has a simple operating procedure and produces immediate results, and the electrochemical method,^11,12^ which is easy to implement and allows continuous measurement. However, DPD-based methods are difficult to use for continuous measurements and can also be disturbed by turbid and natural organic coloring components.^13^ The disadvantage of the electrochemical methods is that the detection electrodes (precious metals such as Pt and Au) used in these methods are prone to surface poisoning (manganese salt coating) and proceed to form electrochemically active surface oxides, which destabilize the signals.^14^

More recently, graphene-based sensors employing Schottky diodes^15^ or solution-gated field-effect transistors^16^ have been reported to demonstrate high sensitivity to free chlorine, which means they are suitable for rapid, real-time, and sensitive sensing and have the potential to solve the challenges mentioned above. Angizi *et al.* demonstrated that graphene/n-Si Schottky junctions modified with 1-aminopyrene exhibit high sensitivity to free chlorine, with a detection limit of 3 ppb.^15^ This heightened sensitivity is attributed to the oxidizing agent, free chlorine, which acts as a p-dopant on graphene to significantly change the carriers in graphene with slight variations in free chlorine concentration. Solution-gated graphene field-effect transistors (GFETs), in which the gate and graphene channels are separated by an electrolytic solution instead of a dielectric insulator, operate with high transconductance based on electric fields generated across the electric double-layers formed at the electrolyte/graphene channel interface. As with ordinary FETs, the channel conductance of solution-gated GFETs can be modulated by the gate voltage *via* field-effect doping. Xiong *et al.* demonstrated solution-gated GFETs for measuring free chlorine in tap water samples.^16^ These GFETs employed Au gate electrodes and the source–drain current was modulated according to the potential change of the Au gate electrode, which follows the Nernst equation in response to the concentration of free chlorine. This indicates that gate electrodes with suitable electrochemical properties are vital for sensing free chlorine.

In this paper, we focused on the gate electrode of the GFET for free chlorine sensing and investigated its effect on the sensing performance. Ultimately, we aimed to improve the performance of free chlorine sensors using solution-gated GFET. In particular, to investigate the effect of the electrochemical properties, including the electric double-layer capacitance, potential window in aqueous solutions, and surface redox species, we utilized graphene and boron-doped diamond (BDD) as gate electrodes for solution-gated GFETs. Graphene deposited using chemical vapor deposition (CVD) has inactive surface electrochemically inactive, similar to the basal plane of highly ordered pyrolytic graphite (HOPG), compared with other carbon-based materials such as glassy carbon.^17^ BDD with boron-to-carbon (B/C) ratios of 1% exhibit metal-like conductivity while maintaining the inherent characteristics of diamond such as physical strength and chemical stability^18^ and, therefore, can be utilized as electrodes for electrochemical analysis and water treatment. Graphene and BDD electrodes exhibit low double-layer capacitances and wide potential windows in aqueous solutions compared with those of Au electrodes because they have fewer adsorption sites on their surfaces. Therefore, electrochemically active species are also scarcely formed on the surfaces of graphene and BDD electrodes. In contrast, the electrochemically active surface oxide formed on metal electrodes, including Au and Pt, detrimentally affects the electrochemical sensors of free chlorine.^19,20^ These surface oxides lead to variations in the amperometric responses and have to be removed using mechanical or electrochemical polishing.^21^

Here, we report that the electric double-layer capacitance is critical for the shift in the Dirac point voltage of transfer curves of GFETs with the free chlorine concentration. We also demonstrate that GFETs can detect low concentrations of free chlorine using graphene and BDD as gate electrodes, which are free of surface redox species.

## 2 Experimental

### 2.1 Preparation of graphene channel and gate electrodes

**Fig. 1:**
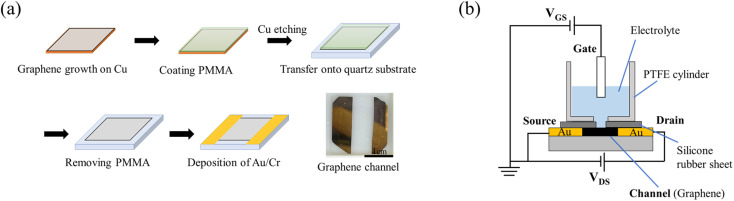
(a) Fabrication of graphene film with channels. (b) Schematic diagram of a solution-gated GFET.

Graphene films were prepared as shown in Fig. 1(a).^22^ Single-layer graphene films were grown on a Cu substrate (35 μm thick, Graphene Platform Corp., Tokyo, Japan) at 1000 °C by CVD using a gas mixture consisting of CH_4_ (20 sccm) and H_2_ (2 sccm) as the reaction source. A thin film of polymethyl methacrylate (PMMA) was spin-coated (2500 rpm, 60 s) onto the graphene, and the graphene film deposited on the reverse side of the copper substrate was removed by oxygen plasma. The PMMA/graphene/Cu was allowed to float in a 0.5 M iron nitrate aqueous solution for 5 hours to etch (remove) the Cu substrate, whereupon the PMMA/graphene was washed six times with DI water. The graphene/PMMA film was then transferred onto a quartz glass substrate (20 mm × 20 mm × 1 mm), and the PMMA on the graphene film was removed by immersion in acetone overnight. After allowing the transferred graphene to dry, Cr/Au (10 nm/40 nm) electrodes were evaporated onto the substrate as source (S) and drain (D) to create a graphene channel. The fabricated graphene channels were designed to have a length of 7.5 mm and a width of 15 mm.

The assembly comprising the solution-gated GFET is illustrated in Fig. 1(b). The GFET was assembled as follows. A silicone rubber sheet with a hole was placed on the graphene channel to prevent the electrolyte solution from coming into contact with the source and drain electrodes. A polytetrafluoroethylene (PTFE) cylinder was mounted on the silicone rubber sheet to contain the electrolyte solution and form the interface between the graphene channel and the electrolyte. Finally, a gate electrode was inserted into the electrolyte solution to complete the fabrication of the solution-gated GFET.

The graphene film used as the gate electrode was fabricated similarly to the process described above but has an Au/Cr contact electrode on one side. The boron-doped diamond (BDD) films used as the gate electrode were deposited on silicon wafers using a microwave plasma-assisted chemical vapor deposition (MPCVD) method with methane and trimethyl boron as the sources of carbon and boron, respectively, at a B/C ratio of 1%.^18,23^ The BDD films were evaluated by Raman spectroscopy (Fig. S1†).^24^ The Au films used as the gate electrode were fabricated by vacuum evaporation with Cr interlayer on SiO_2_/Si substrate (Cr 10 nm/Au 100 nm). These gate electrodes were insulated by an acryl resin coating except for the area used as the electrode (0.75 cm^2^), unless otherwise noted.

### 2.2 Characterization and electrochemical measurements with solution-gated graphene field-effect transistors

The device performance, including the transfer curves (drain–source current *I*_DS_*versus* gate-source voltage *V*_GS_), was characterized using a semiconductor device parameter analyzer (B1500A, Keysight). The transfer curves were recorded at a fixed drain-source voltage (*V*_DS_ = 0.1 V) and by varying the gate-source voltage (*V*_GS_ = 0–1 V) at a sweeping rate of 0.01 V s^−1^. Solutions of free chlorine were freshly prepared by diluting sodium hypochlorite pentahydrate, NaClO·5H_2_O (Tokyo Chemical Industry Co. Ltd, Japan), in DI water and their NaClO concentrations were standardized using a photometric free chlorine meter (AQUAB AQ-202P, SIBATA Scientific Technology Ltd, Japan). Phosphate buffer solution (PBS) was prepared with sodium dihydrogen phosphate dihydrate and disodium hydrogen phosphate (FUJIFILM Wako Pure Chemical Corp., Japan). The electrochemical properties were evaluated by cyclic voltammetry and electrochemical impedance spectroscopy measurements using an Autolab PGSTAT204 potentiostat/galvanostat system equipped with an impedance analyzer FRA32M (Metrohm, Utrecht, The Netherlands).

## 3 Results and discussion

### 3.1 Evaluation of p-type doping effect induced by free chlorine

**Fig. 2:**
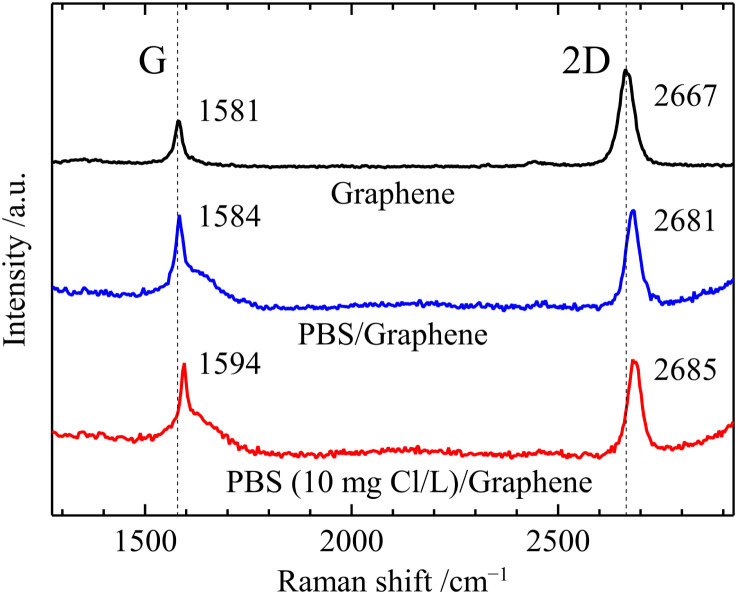
Raman spectrum of graphene/quartz glass, 0.1 M PBS (pH 7)/graphene/quartz glass, and 10 ppm free chlorine-contained 0.1 M PBS (pH 7)/graphene/quartz glass.

In this study, the GFET-based free chlorine sensor operates by the doping effect of free chlorine which deprives graphene of electrons due to its high oxidation ability. First, we performed Raman spectroscopy of graphene in contact with the solution to investigate the doping effect of free chlorine. Fig. 2 shows Raman spectra of graphene before and after the solution contact. The prepared graphene film on a quartz substrate showed the two intensive Raman bands, the G band at 1581 cm^−1^ and the 2D band at 2667 cm^−1^. The intensity ratio of the 2D and G peaks (2D/G) is 2.4, and the 2D peak with a full width at half maximum (FWHM_2D_) value of 42 cm^−1^ can be fitted by a single Lorentzian curve, which is the characteristic signature of single-layer graphene.^25^ The G band originates from a first-order one-phonon scattering process and the 2D from a second-order two-phonon intervalley scattering process, both of which are typical of all sp^2^ carbon materials.^25^ The Raman frequencies of the G (*ω*_G_) and 2D (*ω*_2D_) bands of graphene are influenced by the strain and doping induced by the substrate and environment, and their effects can be assessed based on a correlative analysis of the G and 2D peaks frequencies.^25–27^ The Raman spectra of graphene in contact with the solution were measured with a drop of solution and cover glass on top. In the Raman spectra, the broad peak at the base of the G peak at 1600 cm^−1^ and the tail at higher wavelengths than 3000 cm^−1^ are water-derived signals.^26^ The Raman spectrum of graphene in contact with 0.1 M PBS (pH 7) showed a large blueshift of the 2D peak (Δ*ω*_2D_ = 14 cm^−1^) while the blueshift of the G peak was slight (Δ*ω*_G_ = 3 cm^−1^). Such changes in *ω*_G_ and *ω*_2D_ are caused by an increase in compressive strain rather than charge doping,^27^ and can be attributed to an increase in hydrostatic compressive strain due to the change from air to aqueous solution at the graphene surface. In contrast to these changes in 2D and G, a slight blueshift in the 2D peak (Δ*ω*_2D_ = 4 cm^−1^) and a large blueshift in the G peak (Δ*ω*_G_ = 10 cm^−1^) were observed by replacing the 0.1 M PBS with PBS containing 10 ppm free chlorine between the cover glass and graphene. This is consistent with the change in Raman spectra caused by graphene being p-doped without change in strain,^26^ indicating a significant increase in the carrier density of graphene induced by free chlorine. In line with our study, previous research on tetracyanoethylene (TCNE)-adsorbed graphene has similarly reported shifts in the frequency of G-band and 2D-band due to p-doping.^28^ The p-type doping induced by free chlorine results from the following electrochemical reactions (1) and (2) occur at the graphene surface.

1 ClO^−^ + H_2_O + 2e^−^ → Cl^−^ + 2OH^−^, *E*_0_ = 0.81 V

2 HClO + H^+^ + 2e^−^ → Cl^−^ +H_2_O, *E*_0_ = 1.40 V

where standard redox potential *E*_0_ is measured against standard hydrogen electrode at concentration of 1 mol L^−1^, 1 atmosphere pressure, and 25 °C.^29^

In the reactions in eqn (1) and (2), electrons are withdrawn from the valence band of graphene by the hypochlorite ions (ClO^−^) and hypochlorous acid (HClO) because of the high redox potential. This means the electrode equilibrium potential of graphene shifts to a positive direction in the electrochemical scale. As a consequence of the electrochemical reactions at the graphene surface, the hole and the corresponding anions to be electrically balanced, remain in the graphene and in the solution near the graphene surface, respectively, thus lowering the Fermi level of graphene and increasing the carrier density. The increase in carrier density was confirmed by Hall effect measurements which were performed before and after contact with the solution containing free chlorine. The graphene samples for the Hall effect measurements were patterned with a van der Pauw geometry by photolithography (Fig. S3†). Drops of 0.1 M PBS hardly changed the carrier density and sheet resistance, but after drops of 0.1 M PBS containing 10 ppm free chlorine, the carrier density increased by 34% and sheet resistance decreased by 11% (Table S1†). These observations clearly show that the presence of free chlorine on the graphene surface results in electron withdrawal, leading to an increase in the number of holes in the graphene. Such doping, called surface transfer doping or electrochemical doping, occurs on the surface of two-dimensional materials such as graphene^28,30,31^ or semiconductors such as diamond,^32,33^ resulting in significant changes in electronic states.^34^

The degree of doping in the graphene depends on the redox potential and the ease with which the reaction occurs and should be reflected in the equilibrium electrode potential. In other words, the higher the equilibrium electrode potential, the lower the Fermi level of graphene.

### 3.2 Measurement of free chlorine using the Au gate electrode

**Fig. 3:**
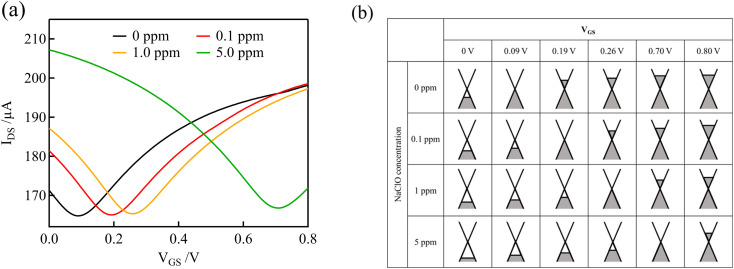
(a) Transfer curves of GFETs with Au gate electrodes at various free chlorine concentrations in 0.1 M PBS (pH 7). (b) Schematic representation of the electronic state of graphene at various NaClO concentrations and *V*_GS_ levels.

Fig. 3(a) shows the transfer curves (*I*_DS_*versus V*_GS_) measured using the graphene channel and Au gate electrode. All the transfer curves show typical ambipolar behavior, indicating that the type of charge carrier in graphene can be continuously modulated from holes to electrons by the field effect.

Fig. 3(b) shows schematic representations of the electronic states of graphene at various NaClO concentrations and *V*_GS_ levels, which correspond to the curves in Fig. 3(a). Graphene has a unique linear band structure referred to as the Dirac cone, in which the valence and conduction bands cross at a point at which no band gap exists. At this point, known as the Dirac point, the carriers in graphene are at a minimum, and the current flowing through the graphene channel reaches a minimum.^35^ Noting the transfer curve when the NaClO concentration is 0 ppm, the graphene channel current is at a minimum at *V*_GS_ = 0.09 V, which corresponds to the Dirac point. When *V*_GS_ < 0.09 V, the Fermi level of graphene is within the valence band, and the hole concentration and current in the graphene channel decrease in response to an increase in *V*_GS_. Conversely, upon application of *V*_GS_ > 0.09 V, the Fermi level of graphene resides in the conduction band, and the current in the graphene channel increases with increasing *V*_GS_ because the electron concentration increases.

As the free chlorine concentration increased, the transfer curve shifted in the positive direction of *V*_GS_, and the current in the graphene channel *I*_DS_ at *V*_GS_ = 0 V increased, as shown in Fig. 3(a). Noting the change in the Fermi level of graphene when *V*_GS_ = 0 V in Fig. 3(b), the Fermi level shifts downward as the NaClO concentration increases. Thus, *V*_DP_, the gate voltage required for the Fermi level to reach the Dirac point, increases with increasing NaClO concentration. The *V*_DP_ at each NaClO concentration was 0.09 V, 0.19 V, 0.26 V, and 0.70 V for 0, 0.1, 1, and 5 ppm, respectively. The change in *V*_DP_, that is, the change in the Fermi level of graphene, is caused by the redox reactions of free chlorine on the graphene channel, as indicated in the previous section.^16^ This means the electrode equilibrium potential of graphene shifts to a positive direction in the electrochemical scale with an increase in free chlorine concentration according to the Nernst equation:3
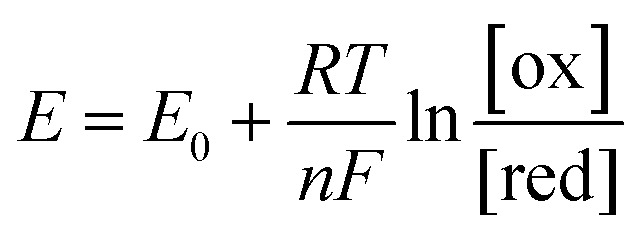
where *R* is the gas constant, *T* is the temperature in Kelvin, *F* is Faraday's constant, *n* is the number of electrons, and [ox] and [red] denote the concentrations of the oxidant and reductant species, respectively.

As indicated by the Nernst equation, the electrode potential depends on the concentration of oxidized and reduced forms of the redox. Therefore, as the free chlorine concentration increases, the Fermi level of graphene shifts downwards because the electrode potential becomes more positive. The increase in the magnitude of graphene doping by elevating the concentration of such redox-active compounds has been reported with other molecules as well. D. J. Late reported that by increasing the concentration of the electron acceptor molecule TCNE, spin-coated onto the surface of a monolayer graphene transistor, graphene tends to shift towards a more p-type doping.^30^

In solution-gated GFET devices, the gate electrode is also exposed to the electrolytic solution as well as the channel. This means that the reactions described in eqn (1) and (2) occur not only on the surface of the graphene channel but also on that of the gate electrode. As a result of these reactions, the potential of the gate electrode also changes based on the Nernst equation.

Here, we consider the transfer curve and Fermi level at the beginning of the measurement. The initial voltage of 0 V at the beginning of the measurement corresponds to the channel and gate being at the same potential. Because the channel and gate potentials follow the Nernst equation, the potential at the start of the measurement (*i.e.*, *V*_GS_ = 0) should be determined from the concentration of NaClO. In fact, as the concentration of free chlorine was increased, the potential *vs.* Ag/AgCl at the start became more positive, resulting in a larger Dirac point voltage. On the other hand, when Ag/AgCl was used as the gate electrode, the potential of the graphene channel was determined by the potential of Ag/AgCl, which eliminated the doping effect of free chlorine on the graphene channel. In this case, the transfer curve was not shifted by changing the free chlorine concentration (Fig. S2†), and this is consistent with a previous study.^16^ Thus, the potential of the gate electrode is a critical parameter for the detection of free chlorine using a solution gated GFET.

### 3.3 Effect of electric double-layer capacitance at the interface between the gate electrode and the solution

The results in Section 3.2 indicate that the potential applied to the gate electrode is vital for the detection of free chlorine. We focused on the gate electrode to increase the shift in the transfer curves. The *V*_GS_ discussed above is distributed over the two electric double layers, as expressed by eqn (4).4
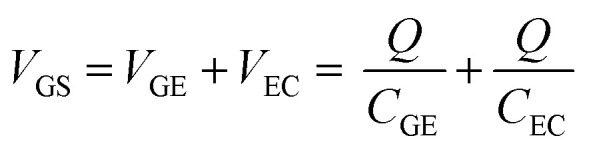
where *V*_GE_ and *V*_EC_ are the voltages across the electric double layers formed at the gate electrode/electrolyte and electrolyte/channel interfaces, respectively; *C*_GE_ and *C*_EC_ are the electric double-layer capacitances at the gate electrode/electrolyte and electrolyte/channel interfaces, respectively; and *Q* is the charge stored in the electric double layers. The charge *Q* stored in the two electric double layers is the same because the solution is electrostatically neutral. Therefore, the distribution of *V*_GE_ and *V*_EC_ should be determined by the ratio of the inverse of *C*_GE_ and *C*_EC_. In this experiment, we varied the surface area of the gate electrode to investigate the effect of the electric double layer generated at the gate electrode/electrolyte interface on the transfer curves, because the capacitance is proportional to the electrode area.

**Fig. 4:**
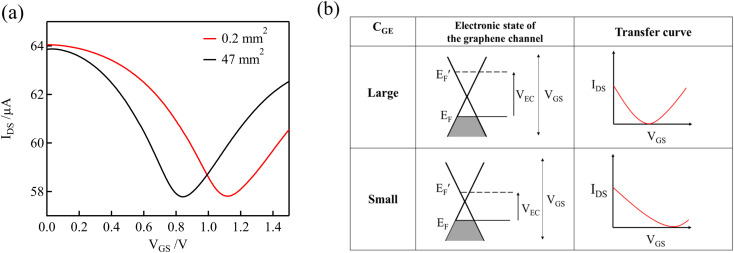
(a) Transfer curves in 0.1 M PBS (pH 7) containing 0.1 mM NaClO using GFET for two different areas of the Au gate electrode. (b) Schematic diagrams of the relation between electronic states and transfer curves, for different double-layer capacitances of the gate electrodes, *C*_GE_.

Fig. 4(a) shows the transfer curves for different areas of the Au gate electrode of the GFET. A comparison of the transfer curves revealed that, for the GFET with a smaller gate electrode area, the Dirac point was observed at higher *V*_GS_. The difference in *V*_DP_ is related to the ratio of the gate voltage distribution in the two electric double layers. The distribution ratio of *V*_GS_ to *V*_EC_ in eqn (4) can be expressed as follows:5
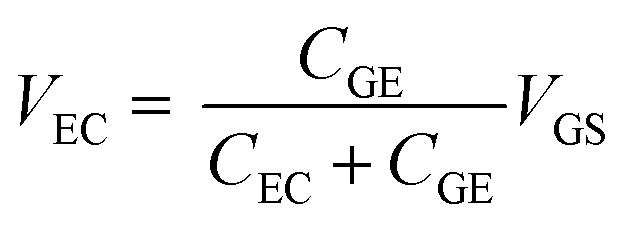


According to eqn (5), when *C*_GE_ is lower, the voltage distribution to the *V*_EC_ is lower. In this case, higher *V*_GS_ is required to effectuate a potential change at the graphene-channel/electrolyte interface, which determines the field effect on the graphene channel. Fig. 4(b) shows the relationship between the electronic state of the graphene channel and the transfer curve when modulating *V*_GS_. When *C*_GE_ is small, the Fermi level of the graphene channel cannot be significantly changed by modulating *V*_GS_ because the voltage distribution to *V*_EC_ is small. As a result, the Dirac point is observed at higher *V*_GS_ for GFETs with a smaller gate electrode area.

As described in Section 3.2, the degree of p-doping, or the Fermi level, of the graphene channel changes depending on the free chlorine concentration. In a GFET with a low *C*_GE_, even if the graphene channel is slightly p-type doped by a slight change in the NaClO concentration, a high gate voltage *V*_GS_ is required to modify the potential of the graphene channel side. Consequently, the shift in the Dirac point on the transfer curve becomes large, even for a small change in the NaClO concentration. In other words, even minute changes in the concentration can be detected.

### 3.4 Electrochemical properties of the gate electrodes

Because the electric double-layer capacitance of the gate electrode is an essential parameter in solution-gated GFET, other electrode materials with lower electric double-layer capacitance than Au, such as graphene and BDD, were used as gate electrodes to improve the sensitivity. The lower electric double-layer capacitances of the graphene and BDD electrodes are considered to be due to the lower density of states near the Fermi level.^36–38^ The electrochemical properties of these electrodes were evaluated before using them as the gate electrodes of the solution-gated GFETs.

**Fig. 5:**
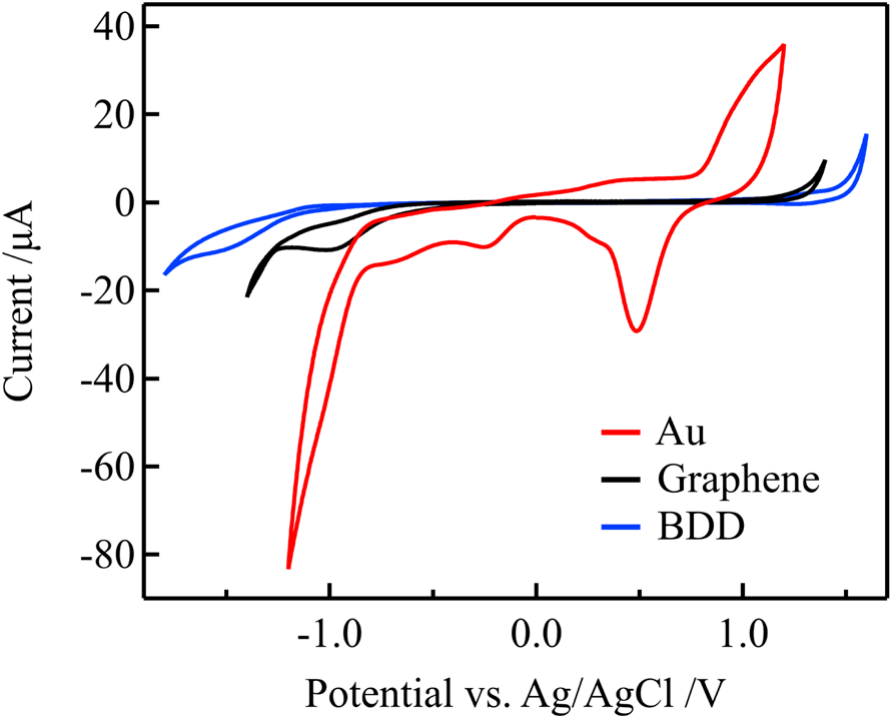
CVs for in 0.1 M PBS (pH 7) for the Au, graphene, and BDD electrodes at a scan rate of 0.1 V s^−1^.

Fig. 5 shows the cyclic voltammograms (CVs) that were acquired using the Au, graphene, and BDD electrodes in a 0.1 M PBS with pH 7. Compared with the Au electrode, the graphene and BDD electrodes have more expansive potential windows, an indicator of electrochemical stability. In addition, the considerably lower current in the windows represents lower double-layer capacitances. Furthermore, redox waves associated with the surface functional groups were not observed in the potential window. In contrast to the graphene and BDD electrodes, the CV of the Au electrode exhibits oxidation and reduction peaks of Au in the range of 0.3 to 1.1 V that were attributed to the formation of electrochemically active surface oxide.^39^ These results suggest that the graphene and BDD electrodes have more stable electrode surfaces than the Au electrodes.

Using electrochemical impedance spectroscopy, the electrical double-layer capacitance of the Au, graphene, and BDD electrodes in 0.1 M PBS was estimated as 27.3, 1.65, and 2.68 μF cm^−2^, respectively. Therefore, the graphene and BDD gate electrodes are expected to require a higher gate voltage compared to the Au gate electrodes for changing the potential on the graphene channel in solution-gated GFETs. As a result, the transfer curves should be more significantly shifted with changes in the free chlorine concentration when graphene and BDD are used as gate electrodes.

### 3.5 Dependence of transfer curves on free chlorine concentration

The graphene and BDD electrodes, which had a lower electrical double-layer capacitance than the Au gate electrodes, were used as gate electrodes of the solution-gated GFET to record the transfer curves in solutions containing various concentrations of free chlorine. For the measurements of solutions with various concentrations of free chlorine, the concentration of free chlorine was adjusted by adding an appropriate volume of 0.1 M PBS containing 100 ppm of free chlorine, stepwisely increasing the concentration while measuring the transfer curve at each concentration. After each measurement, the channel and gate potentials were reset by connecting the gate electrode and source terminals to the Ag/AgCl electrode inserted in the solution. Free chlorine was then added to the solution and stirred, and allowed to stand for 1 minute before starting the next measurement (the transfer curves in Fig. 3(a) were also measured with the same procedure).

**Fig. 6:**
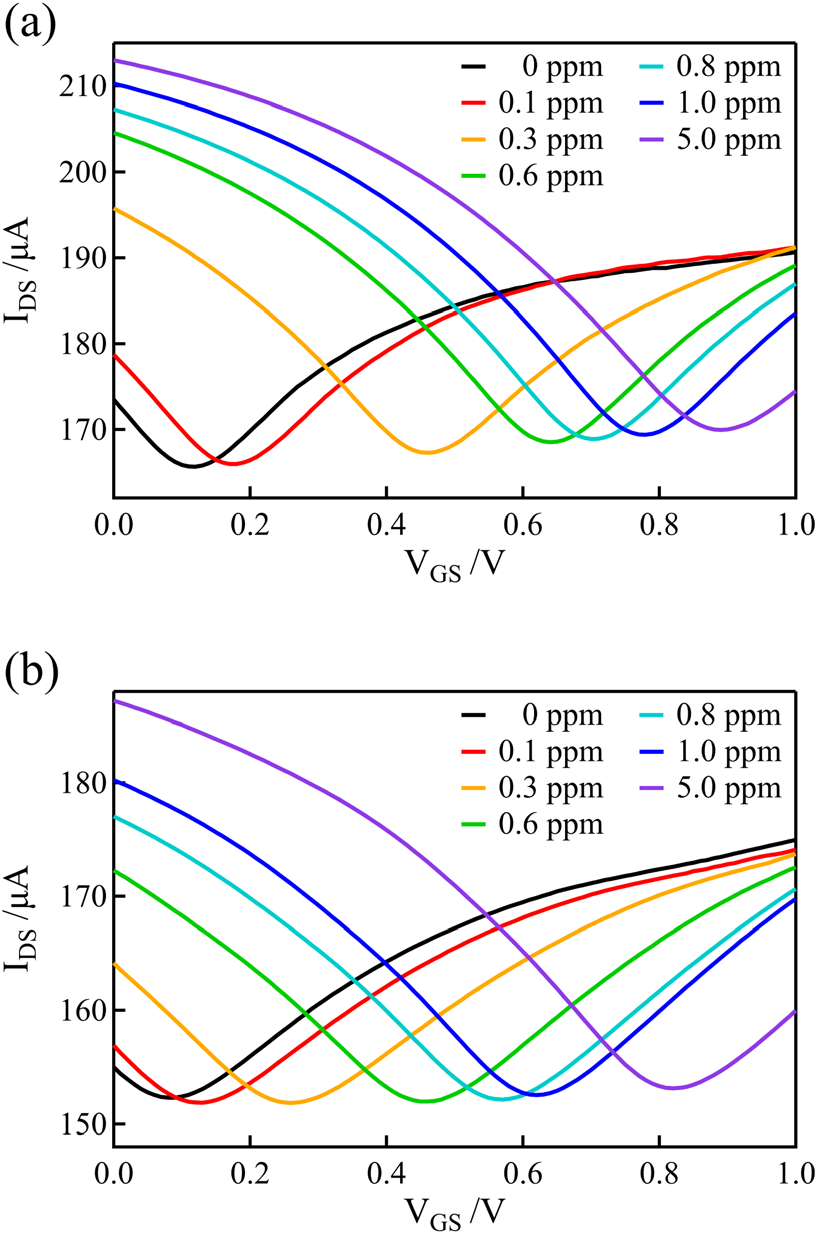
Transfer curves recorded in 0.1 M PBS for various free chlorine concentrations in GFETs with (a) graphene and (b) BDD gate electrodes. The concentrations listed in the legend indicate the concentration of free chlorine in the solution.

Fig. 6(a) and (b) show the transfer curves using GFET with graphene and BDD gate electrodes, respectively. Distinct shifts in the transfer curves were observed as the concentration of free chlorine increased. These shifts were observed even at low concentrations below 1 ppm, which is within the range of ordinary free chlorine concentrations in tap water.

**Fig. 7:**
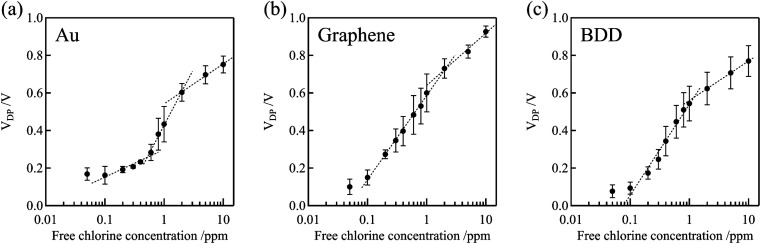
Relationship between *V*_DP_ and free chlorine concentration for the Au, graphene, and BDD gate electrodes. The *V*_DP_ of the blank solution (0 ppm) for Au, graphene, and BDD gate electrodes were 0.090 ± 0.012, 0.12 ± 0.032, and 0.057 ± 0.028 V, respectively.

Fig. 7 shows the dependence of the Dirac point voltage *V*_DP_ on the free chlorine concentration for the Au, graphene, and BDD gate electrodes. The average of three measurements performed with each gate electrode was plotted and the standard error was used as the error bar. An increase in *V*_DP_, proportional to the logarithm of free chlorine concentration, was observed in accordance with the Nernst equation, despite the presence of high error bars. One plausible explanation for the wide variation in the error bars is the inherent variability among individual samples, which were measured across multiple channels. Notably, compared with the Au gate electrode, the shift in *V*_DP_ for the graphene and BDD gate electrodes was larger and more linear with respect to the logarithm of free chlorine concentration at low concentrations range (0.1–0.6 ppm). This is not only attributable to the difference in the electrical double-layer capacitance but also to the electrochemically active surface oxides on the Au electrode. The surface oxides cause the potential of the Au electrode to be the mixed potential as a combination of the redox reactions of both the free chlorine and surface oxides. In the low free chlorine concentration range (below 0.4 ppm), the potential of the Au electrode is likely to be dominated by the surface redox species and is largely independent of changes in the free chlorine concentration. The absence of surface redox reactions at the graphene and BDD electrodes is likely to be responsible for the large shift in the Dirac-point voltage, even in the low-concentration range. For the method to determine the free chlorine concentration based on the Dirac point voltage, the limit of detection was about 0.1–0.2 ppm for BDD and graphene gate electrodes, while about 0.5 ppm for Au gate electrodes, as estimated from these calibration curves. It can be difficult to accurately measure chlorine levels when the concentration is below 0.1 ppm. This is because there are fewer redox reactions to govern the potential, causing the electrode potential to fluctuate more. Additionally, free chlorine is a relatively reactive species and can decrease or even disappear during the measuring process, making it challenging to obtain precise measurements.

In addition to the method of determining concentration from the Dirac point voltage *V*_DP_, free chlorine was also measured by monitoring the drain-source current *I*_DS_ at a specific operating point with the gate voltage *V*_GS_ at 0.1 V (Fig. S4†). As can be expected from the change in the transfer curves, an increase in drain-source current *I*_DS_ was observed with increasing free chlorine concentration. For all gate electrode configurations, a noticeable rise in drain–source current was observed, even when the free chlorine concentration increased from 0 to 10 ppb. This suggests that GFETs possess significant potential for measuring free chlorine with high sensitivity. However, large current fluctuations were observed for solutions below 10 ppb of free chlorine, possibly due to the unstable electrode potential of the channel and gate.

On the other hand, in the high concentration range (above 1 ppm), the change in *V*_DP_ was similar for all the electrodes and was smaller compared with that in the concentration range of 0.5 to 1.0 ppm. This is because the starting potential (*i.e.*, equilibrium potential) shifts toward the positive side at high concentrations according to the Nernst equation, and the potential of the gate electrode immediately reaches the positive limit of the potential window as the gate voltage increases, resulting in a larger gate voltage being distributed to the electric double layer at the graphene channel–electrolyte interface. According to eqn (5), a lower capacitance of the electric double layer at the gate electrode–electrolyte interface lowers the distribution rate to the electric double layer at the graphene channel–electrolyte interface; however, if the potential of the gate electrode cannot be changed, all the changes in the gate voltage *V*_GS_ should be applied to the voltage change in the double layer on the graphene channel. Therefore, regardless of the electric double-layer capacitance, the change in *V*_DP_ with respect to the concentration change became smaller for all gate electrodes. As shown in Fig. 5, it can be observed that oxidation reaction initiated from approximately 1 V *vs.* Ag/AgCl while their current was quite low at graphene and BDD electrodes. As this current arises from water oxidation reactions, it will continue to flow as long as the potential is maintained, in contrast to the charging current and the faradaic current associated with the surface redox group. Hence, during measuring transfer curves, after the gate potential reaches the positive limit of the potential window to start water oxidation, the gate electrode remains the potential until the channel potential reaches a potential where a reduction reaction starts on the graphene channel. In Fig. S2,† the Dirac point is observed at 0.05 V of Ag/AgCl gate relative to the source, which means that the Dirac point potential of the graphene channel is located at −0.05 V *vs.* Ag/AgCl in these measurements. As the concentration of free chlorine increased, the equilibrium potential shifted to the positive side, and the gate potential reached the potential limit around 1 V before the channel potential reached −0.05 V during increasing *V*_GS_. This can be considered to have resulted in a smaller change in the Dirac point voltage in the high concentration range. At a free chlorine concentration of 10 ppm, the equilibrium potentials at the start of the measurements using Au, graphene, and BDD gate electrodes were 0.70 V, 0.67 V, and 0.52 V *vs.* Ag/AgCl, respectively.

In these measurements, the Dirac point was observed on all the transfer curves. This is considered to be because the potential limit of the graphene channel is not on the positive side of the Dirac point potential in the electrochemical scale. If the reduction reaction occurs on the graphene channel before the channel potential reaches the Dirac point, a leakage current would flow, and the transfer curve would be disturbed. In these measurements, the first reduction reaction that occurs on the graphene channel is the reduction of free chlorine, which starts at approximately 0 V *vs.* Ag/AgCl, close to the potential at which the Dirac point is observed (Fig. S5†). Therefore, it can be considered that a slight reduction reaction of free chlorine occurs on the channel when the Dirac point is observed, although the amount is small because the free chlorine concentration is low. In fact, the onset of leakage current increase roughly coincides with the Dirac point voltage (Fig. S6†). The concentration of free chlorine at which the leakage current becomes non-negligible for the source–drain current is dependent upon the geometry of the channel and the drain voltage. In the present experimental setup, under 10 ppm conditions, the drain current recorded 170 μA, accompanied by a corresponding leakage current of 0.22 μA at the Dirac point voltage. Consequently, the leakage current proved negligible in comparison to the drain current and its variations, with no significant impact observed on the transfer curves.

However, the transfer curves are asymmetrical shapes, exhibiting smaller transconductance (*g*_m_ = |Δ*I*/Δ*V*|) values for higher gate voltages than *V*_DP_ (Fig S7†). The diminished increase in drain–source current in the n-channel operation is derived from the decreasing distribution of gate voltage to the electric double layer at the channel/electrolyte interface due to the reduction reactions on the channel. As well as the case of gate electrodes under high concentrations of free chlorine, the change in channel potential is constrained until the gate potential reaches the potential at which the oxidation reaction occurs. Leakage current may also cause the suppression of an increase in drain current under n-channel operation because it induces the voltage drops within the electrolyte solution, hindering the increase in the voltage across the double layer at the electrolyte–channel interface. Importantly, for typical chlorine concentrations in drinking water, the channel potential reaches the Dirac point voltage before reaching the potential where the reaction of free chlorine becomes significant. Therefore, in this measurement, it can be confidently stated that the Dirac point voltage is invariably obtained.

Thus, the method using the Dirac point shift with solution-gated GFETs was demonstrated to be suitable for the measurement of the free chlorine concentration, particularly in the low-concentration range which is the typical concentration range in drinking water.

## 4 Conclusion

In this study, we elucidated the intricate role played by the gate electrode in solution-gated GFETs for free chlorine sensing. The remarkable sensitivity of solution-gated GFETs to free chlorine arises from the intricate interplay between the electrode potential of the gate electrodes and graphene channels, governed by the Nernst equation. Specifically, when graphene serves as a channel or gate electrode, the alteration in electrode potential aligns fundamentally with the variation in p-doping induced by free chlorine. Consequently, the electrochemical properties of the gate electrode exert a profound influence on the transfer curves for free chlorine sensing. Notably, in GFETs featuring gate electrodes with low electric double-layer capacitance, even minute concentrations of free chlorine demand a substantial gate voltage *V*_GS_ to induce a potential change in the graphene channel, resulting in a significant shift in the Dirac point due to the lower voltage distributed to the double-layer at the channel. Indeed, in practice, GFETs with gate electrodes composed of graphene and BDD, both exhibiting low electric double-layer capacitance, demonstrated superior sensor sensitivity to free chlorine compared to an Au gate electrode. These results also indicate the preference for gate electrode materials that are less prone to forming redox species on the surface, as the gate electrode potential is primarily governed by the concentration of free chlorine.

Practical challenges persist in sensor applications for continuous measurements, particularly regarding the durability of the graphene channel. Continuous and prolonged contact of graphene, being a monatomic layer material, with a liquid is deemed impractical for long-term use. Hence, we advocate for a sensor structure resembling an extended-gate FET, where direct contact between the graphene channel and the sample is avoided.^40^ In this context, a boron-doped diamond (BDD) electrode emerges as an ideal gate electrode material. Not only does it possess a small double-layer capacitance and resistance to forming surface redox species, but it also offers durability and allows for refresh processes *via* electrochemical cleaning.^23^

Assessing sensor reliability requires considering the influence of external substances. Prior research has confirmed the robustness of GFET-based sensors against electrochemically inert ions and various contaminants.^16^ However, since the method relies on the electrode potential primarily dictated by the free chlorine concentration, the impact of redox species apart from free chlorine becomes more significant. Typically, drinking water contains few electrochemically active species beyond free chlorine and oxygen, minimizing their practical effect on measurements. If interfering redox species are present, enhancing the contribution of free chlorine to the electrode potential can be achieved by modifying the gate electrode surface with free chlorine-sensitive substances like amine groups.^15,41^ This modification stabilizes the electrode potential by providing moderate redox properties to electrodes with inert surfaces like BDD, thereby decreasing potential fluctuations at low free chlorine concentrations and enhancing sensor reproducibility. Moreover, the implementation of a dual-gate configuration using materials with different sensitivities and achieving multi-signal integration could effectively incorporate statistical approaches, such as machine learning, thereby enhancing accuracy and robustness.

In summary, this study provides comprehensive design guidelines for a solution-gated free chlorine sensor, positioning it as a promising tool for the convenient and highly sensitive continuous measurement of free chlorine, particularly in ensuring the safety of drinking water. The outlined considerations, particularly those related to the essential elements of the gate electrode, significantly contribute to the development of an effective measurement method.

## Conflicts of interest

There are no conflicts to declare.

## Supplementary Material

RA-014-D3RA07692J-s001
