# Supplementary material for: Impact of gate electrode on free chlorine sensing performance in solution-gated graphene field-effect transistors
Source: RSC Adv. 2024 Mar 6;14(11):7867–76. doi: 10.1039/d3ra07692j (PMC10915591; doi:10.1039/d3ra07692j)
Supplement: RA-014-D3RA07692J-s001 [file RA-014-D3RA07692J-s001.pdf]

Electronic Supplementary Material (ESI) for RSC Advances.
This journal is © The Royal Society of Chemistry 2024

Supplementary Information

Impact of Gate Electrode on Free Chlorine Sensing Performance in Solution-Gated Graphene Field-Effect Transistors

Masato Sugawara,[a] Takeshi Watanabe,*[a] Yasuaki Einaga[b] and Shinji Koh[a]

[a] *Department of Electrical Engineering and Electronics, College of Science and Engineering, Aoyama Gakuin University, Sagamihara, 252-5258, Japan.*

[b] *Department of Chemistry, Faculty of Science and Technology, Keio University, 223-8522, Japan*

Email: twatanabe@ee.aoyama.ac.jp (T.W.)

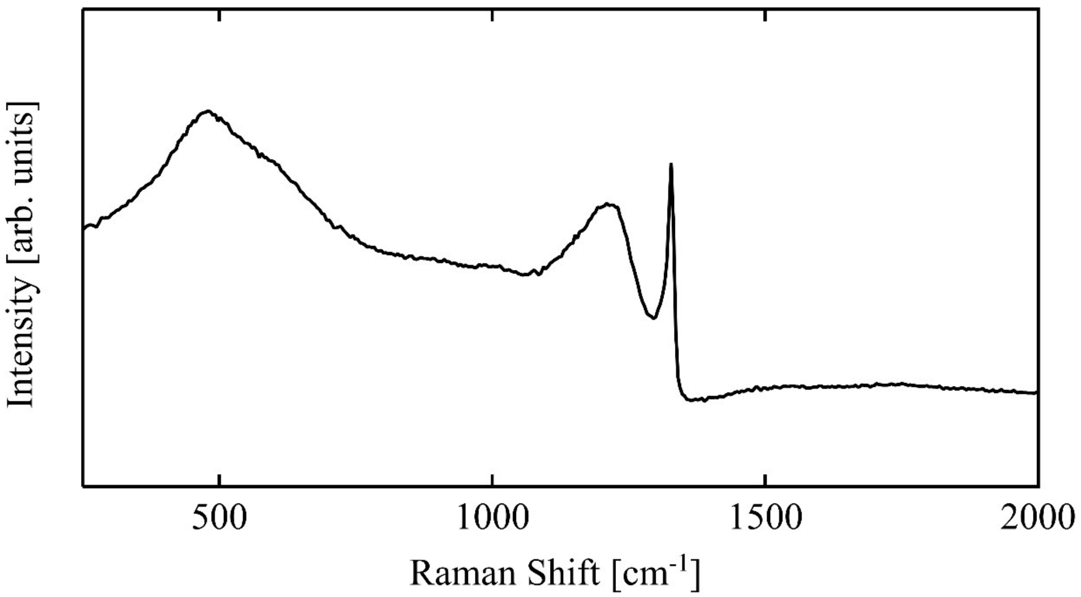

Fig. S1. Raman spectrum of BDD

The Raman spectrum of the BDD film used for the gate electrode of GFET is shown in Fig. S1. The Raman spectrum is typical of diamond films doped with a large amount of boron. The zone-center optical phonon peak for diamond, which is normally observed around 1330 cm^{-1} for intrinsic diamond, was observed at 1326 cm^{-1}. The band centered around 1220 cm^{-1} is generally observed in metallic BDD films and is attributed to the maximum of the phonon density of states (PDOS) in diamond, which appears as a result of relaxation of the wavevector selection rules in the Raman process due to the disorder generated by impurities and/or defects.[1] The band centered around 470 cm^{-1} is considered to be the result of vibration modes of the disordered diamond lattice, although its origin remains unclear.[2] The BDD is almost free from sp^2-bonded carbon because any peaks attributed to amorphous carbon and the G band were not observed.

1 I. I. Vlasov, E. a. Ekimov, a. a. Basov, E. Goovaerts and a. V. Zoteev, 2008, 7.

2 V. Mortet, Z. Vlčková Živcová, A. Taylor, O. Frank, P. Hubík, D. Trémouilles, F. Jomard, J. Barjon and L. Kavan, *Carbon N Y*, 2017, **115**, 279–284.

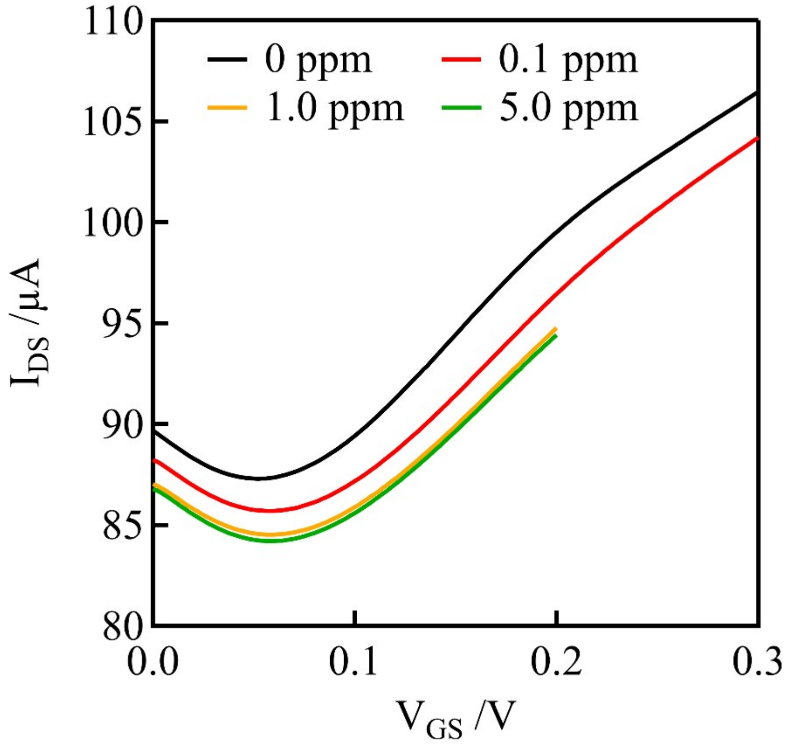

Fig. S2. Transfer curves of GFETs with the Ag/AgCl gate electrode at various free chlorine concentrations.

Figure S2 shows the transfer curves in phosphate buffer solution (PBS) when Ag/AgCl is used as the gate electrode of the graphene field effect transistor, in which case the Dirac point voltage did not shift even as the concentration of free chlorine increased. This is because the potential of the Ag/AgCl electrode depends on the redox potential of the electrode itself rather than the free chlorine.

Table S1 Results of Hall effect measurement of graphene with and without solution contact.

	**Graphene**	**PBS/Graphene**	**PBS (10 ppm-Cl_2)/Graphene**
Carrier density [1/cm^2]	1.45×10^{13}	1.33×10^{13}	1.78×10^{13}
Hall coefficient [m^3/C]	43	47	35
Mobility [cm^2/Vs]	808	883	742
Sheet resistance [Ω/□]	533	530	472

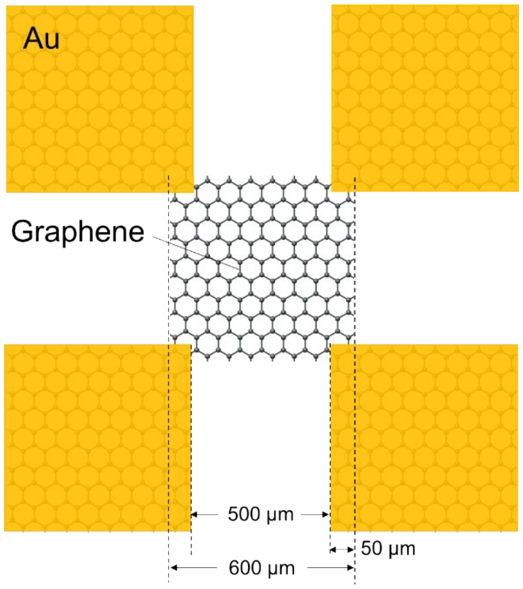

Fig. S3. Design of graphene with van der Pauw geometry for Hall effect measurements.

The graphene samples shown in Fig. S3 were fabricated by photolithography. In the samples, a layer of graphene film is positioned beneath four gold contact electrodes. Although the carrier density decreased slightly when 0.1 M PBS was dropped on the graphene channel, the carrier density increased by 34% and the sheet resistance decreased by 11% after exposure to drops of 0.1 M PBS containing 10 ppm NaClO.

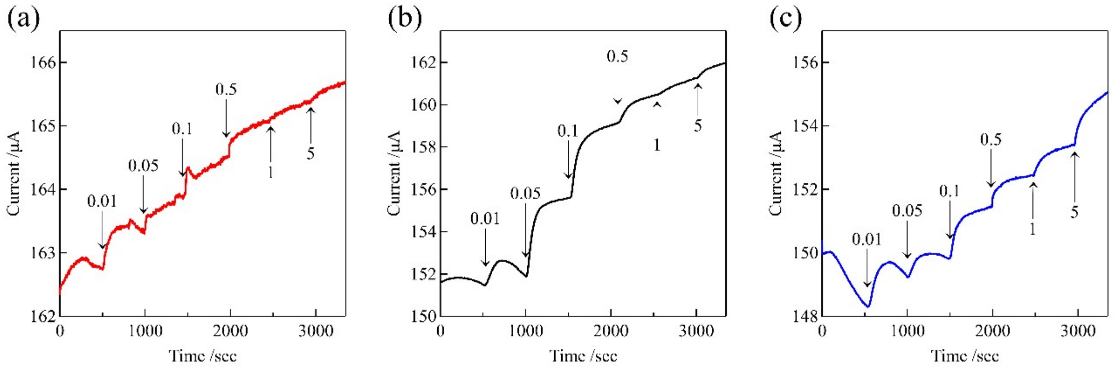

Fig. S4. The channel current response of the solution-gated GFET with (a) Au, (b) graphene, and (c) BDD gate electrodes to an increasing NaClO concentration in 0.1 M PBS measured at $V_G = 0\ V$ and at constant $V_{DS} = 0.01\ V$.

When the concentration of free chlorine is below 10 ppb, the drain current I_{DS} undergoes significant fluctuations. This is because low concentrations of free chlorine or the complete absence thereof destabilize the electrode potential for both the graphene channel and gate electrode. However, even with these fluctuations, the drain current I_{DS} noticeably increases for concentration changes as small as 10 ppb for each of the gate electrodes.

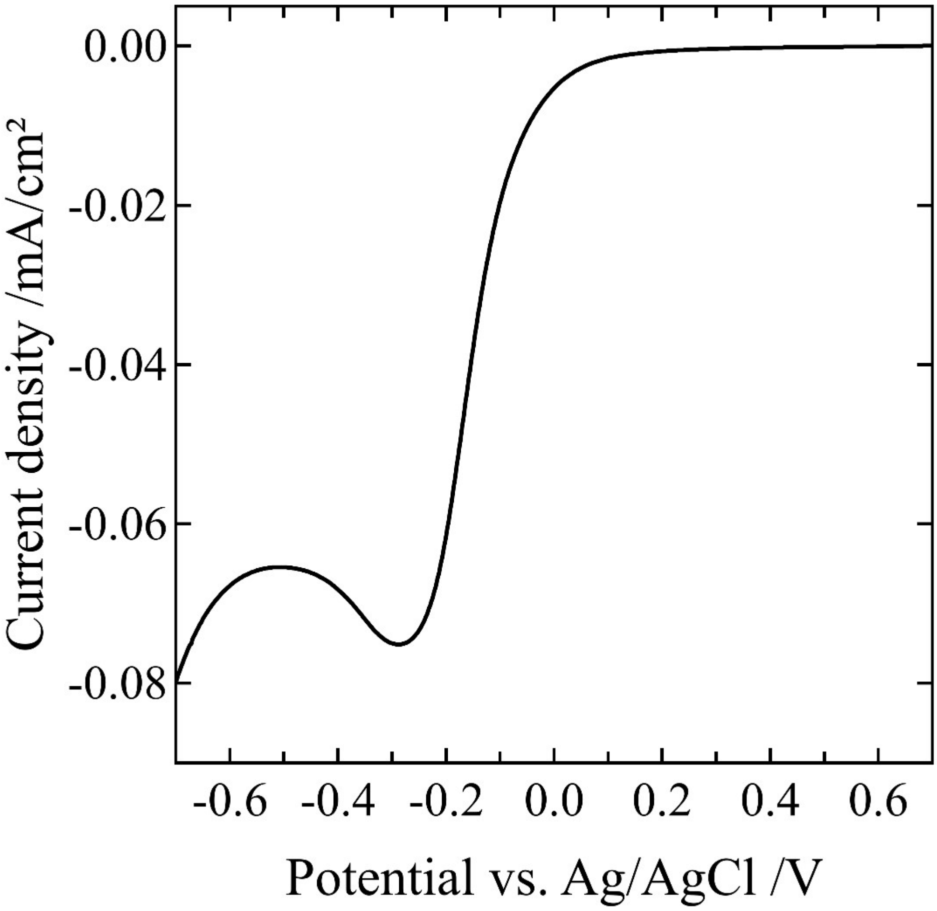

Fig. S5. Linear sweep voltammogram using CVD-grown graphene electrodes in 0.1 M aqueous $NaClO_4$ solution (pH 7.3) containing 1.4 mM NaClO (100 mg-Cl_2/L) with a small amount of $HClO_4$ to adjust the pH.

The reduction peak observed at −0.3 V is attributed to the reduction of hypochlorous acid according to the following reaction.

$$HClO + 2e^- \rightarrow Cl^- + OH^-$$

At potentials more negative than −0.5 V, ClO^- is also reduced along with dissolved oxygen.

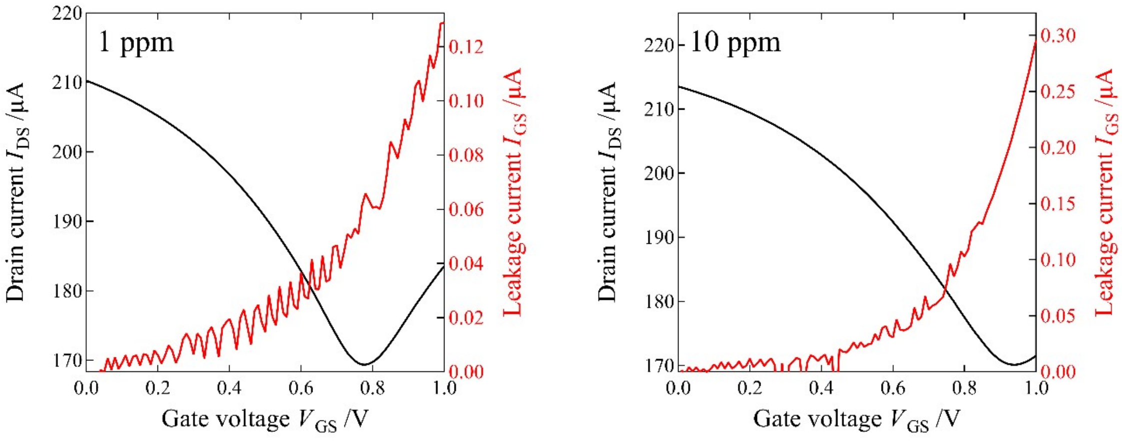

Fig. S6. Transfer curves (black curve) with leakage current (red curve) of the solution-gated GFET with a graphene gate characterized in 0.1 M PBS containing 0.1, 1, and 10 ppm free chlorine.

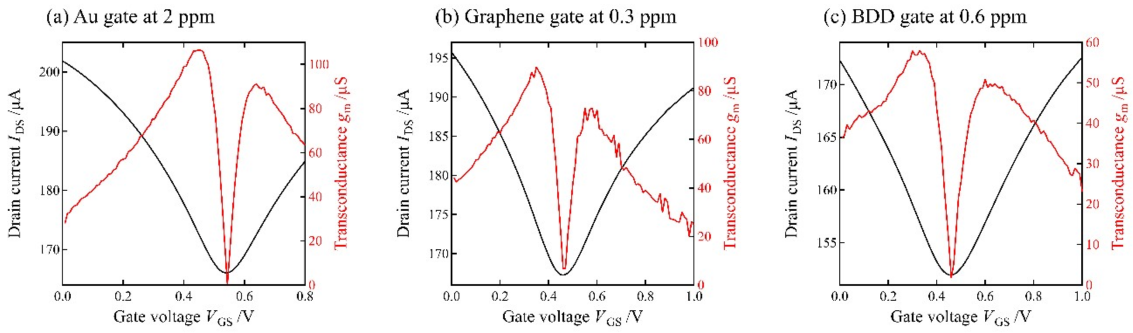

Fig. S7. Transfer curves (black) and transconductance of the solution-gated GFET with (a) Au, (b) graphene, and (c) BDD gates, respectively, characterized in 0.1 M PBS containing free chlorine.
